# Trust-Aware Environmental State Consensus for Smart Agriculture with TEE-Enabled Sensing and Byzantine-Resilient Blockchain Coordination

**DOI:** 10.3390/s26144577

**Published:** 2026-07-19

**Authors:** Lanlan Li, Charles Z. Liu, Kejia Huang, Ruiyi Deng, Majed Abdullah Alrowaily, Mansoor Alghamdi, Ibrahim S. Alkhazi, Malek Alrashidi

**Affiliations:** 1 School of Information Engineering, Chuzhou Polytechnic, Chuzhou 239000, China; lilanlan@chzc.edu.cn; 2 Security TEEs, AlphaNest, Sydney, NSW 2007, Australia; zhenzhong.liu@sydney.edu.au (C.Z.L.); khua0373@uni.sydney.edu.au (K.H.); rden3535@sydney.edu.au (R.D.); 3 School of Electrical and Computer Engineering, University of Sydney, Sydney, NSW 2007, Australia; 4 College of Computer and Information Sciences, Jouf University, Sakaka 72341, Saudi Arabia; 5 Department of Computer Sciences, Applied College, University of Tabuk, Tabuk 47512, Saudi Arabia; malghamdi@ut.edu.sa (M.A.); mqalrashidi@ut.edu.sa (M.A.); 6 Department of Information Technology, College of Computer, Qassim University, Buraydah 51452, Saudi Arabia; i.alkhazi@qu.edu.sa

**Keywords:** trusted execution environment, Byzantine fault tolerance, environmental state consensus, smart agriculture, Internet of Things (IoT), blockchain coordination, edge computing, cyber–physical systems, secure data aggregation, permissioned blockchain

## Abstract

This paper proposes a trust-aware environmental state consensus framework for smart agriculture that integrates TEE-enabled sensing, Byzantine-resilient aggregation, and lightweight blockchain-based state coordination under resource-constrained IoT environments. Unlike conventional IoT systems that treat blockchain as a transactional ledger for directly storing sensor outputs, the proposed framework utilizes blockchain as a state commitment layer that records only validated environmental state transitions. In the proposed architecture, distributed sensor readings are modeled as noisy and potentially adversarial observations of an underlying physical state rather than directly trusted measurements. To establish a reliable trust boundary between physical sensing and distributed coordination, TEE-enabled sensing devices provide authenticated and integrity-protected data outputs before blockchain processing. The TEE component is adopted as a deployed trusted execution anchor rather than a newly designed hardware security mechanism, and its role is to protect sensing-side execution and provide trustworthy inputs for subsequent coordination. Since trusted execution alone cannot guarantee the correctness of sensor observations, a Byzantine-resilient aggregation mechanism is introduced to estimate consistent environmental states under faulty or adversarial sensing conditions. The validated states are then committed through a lightweight permissioned blockchain to provide tamper-evident state finality using a K-confirmation-based commitment mechanism. The proposed framework is implemented and evaluated on a real greenhouse IoT platform with distributed sensing nodes and edge computing devices. Experimental results demonstrate that the proposed approach improves environmental state consistency under varying adversarial conditions while maintaining stable blockchain coordination and resource-aware execution performance.

## 1. Introduction

The reliable acquisition and interpretation of environmental data has become a fundamental requirement for large-scale monitoring and decision-support systems, particularly in scenarios involving distributed sensing infrastructures and sustainability-oriented applications. In this context, data-driven environmental assessment frameworks such as measurement, reporting, and verification (MRV) systems increasingly rely on trustworthy sensing pipelines to ensure consistency and traceability of observed environmental conditions [1,2]. However, in practice, environmental data collected from distributed sources are often affected by noise, missing readings, device heterogeneity, and communication uncertainties, which can significantly undermine the reliability of downstream analysis and state estimation.

To address these challenges, IoT-based sensing infrastructures have been widely adopted for real-time environmental monitoring and automation in agriculture and related cyber–physical systems [3,4,5]. Traditional agricultural systems, however, continue to face inefficiencies and environmental degradation issues [6,7,8], motivating the deployment of distributed sensing and control technologies to enable fine-grained and continuous environmental observation.

In modern smart agriculture systems, IoT sensing is typically integrated within AIoT architectures that jointly consider sensing, communication, and edge computing to support real-time environmental monitoring and decision-making [9,10,11,12,13]. Edge computing further enhances system responsiveness by enabling localized data processing near sensing sources, thereby reducing communication latency and bandwidth consumption [14,15,16]. Nevertheless, practical deployments remain constrained by heterogeneous device capabilities, unreliable wireless communication links, and limited computational resources at edge nodes.

Beyond efficiency considerations, a more fundamental challenge lies in the reliability and consistency of distributed environmental sensing itself. In real-world deployments, sensor observations may be subject to calibration drift, environmental noise, transmission failures, or even malicious manipulation, resulting in inconsistencies between raw observations and the underlying physical environmental state. This motivates the need for robust mechanisms capable of constructing consistent environmental-state representations from distributed and potentially unreliable sensing data, particularly under adversarial or uncertain operating conditions.

Blockchain technology has been widely explored as a tamper-evident infrastructure for decentralized data management [1,2]. However, most existing systems treat blockchain as a transactional ledger, while environmental data remain off-chain and vulnerable before commitment. This creates a gap between blockchain-based integrity guarantees and the physical uncertainty of sensing systems.

A key research gap is therefore the lack of a unified mechanism for constructing a trustworthy environmental state from distributed sensing observations under adversarial conditions. Existing IoT systems focus on data collection, while blockchain systems ensure integrity after data publication. The missing abstraction is an environmental-state consensus mechanism that connects raw sensor observations with a consistent system-level state representation.

Importantly, we distinguish between data integrity and physical truth. Cryptographic mechanisms such as hash commitments and Trusted Execution Environments (TEEs [17,18,19]) can ensure that data have not been tampered with after capture, but they cannot guarantee the correctness, calibration, or representativeness of sensor measurements. The objective of this work is therefore to construct tamper-evident and consensus-driven environmental-state commitments, rather than to recover absolute physical truth.

From a system perspective, blockchain is reinterpreted in this work not as a financial ledger, but as a lightweight coordination layer that records finalized environmental-state commitments derived from distributed sensing agreement. This shift enables blockchain to serve as a state synchronization mechanism for resource-constrained AIoT environments rather than a transaction processing system.

To address these challenges, we propose a trustworthy blockchain-enabled AIoT framework for smart agriculture that integrates TEE-assisted edge computing, Byzantine-robust sensing aggregation, and lightweight blockchain-based state coordination. The proposed framework enables distributed sensors to collaboratively construct a consistent environmental state under uncertainty and adversarial conditions, while ensuring tamper-evident final state commitments.

Unlike prior studies that independently integrate blockchain, Trusted Execution Environments (TEEs), or Byzantine fault tolerance mechanisms (e.g., [20,21,22,23,24,25,26,27,28,29]), this work investigates how these trust mechanisms interact to establish reliable environmental states from distributed IoT sensing observations under adversarial conditions.

It should be noted that the greenhouse sensing infrastructure, embedded edge-intelligence mechanisms, and AI-assisted agricultural analytics adopted in this study were investigated separately in our previous AIoT research [30]. While that work focused on intelligent sensing, embedded edge intelligence, and AI-enabled decision support for smart agriculture, the present study addresses a complementary challenge: establishing trustworthy environmental states from distributed and potentially compromised sensing observations. Consequently, sensing measurements are treated as inputs to the trust framework, enabling the analysis to focus on environmental-state verification, Byzantine-resilient aggregation, trusted execution, and blockchain-assisted state commitment.

Unlike existing studies that assume TEE-based execution environments provide end-to-end trust guarantees [29,31,32], we consider a more realistic threat model in which TEEs can protect execution integrity but cannot prevent malicious, faulty, compromised, or manipulated sensor observations. Consequently, trustworthy environmental-state construction requires coordinated trust across execution, aggregation, and ledger layers rather than relying on a single protection mechanism.

The main contributions of this work are summarized as follows.

- We propose a layered trust-aware architecture for smart agriculture IoT systems that explicitly separates execution trust, sensing reliability, and blockchain state integrity. The proposed architecture provides a system-level framework for understanding how trust information propagates across heterogeneous sensing, edge computing, and distributed coordination layers.
- We design a trust-conditioned environmental-state consensus mechanism that integrates TEE-enabled sensing outputs with Byzantine-resilient aggregation. Instead of assuming that trusted execution guarantees sensing correctness, the proposed mechanism combines execution integrity with adversarial-aware state estimation to mitigate the influence of noisy, faulty, and manipulated sensor observations.
- We establish a lightweight blockchain-based state coordination framework with explicit cross-layer trust interactions. The framework records validated environmental-state commitments rather than raw sensing data and defines how authenticated sensing outputs, robust aggregation, and blockchain finality jointly support trustworthy environmental-state consensus.
- We implement and evaluate the proposed framework on a greenhouse-based smart agriculture prototype equipped with STM32L5 TrustZone-enabled sensing devices and edge computing nodes. Experimental results demonstrate that the proposed framework improves environmental-state consistency and maintains reliable blockchain commitment under benign and adversarial sensing conditions in resource-constrained IoT environments.

In this work, TEE is not introduced as a newly designed hardware security mechanism. Instead, it serves as a deployed trusted execution anchor that provides authenticated and integrity-protected sensing outputs for the proposed blockchain coordination framework.

From a system-design perspective, the proposed framework does not introduce new cryptographic primitives, blockchain protocols, or Byzantine consensus algorithms. Instead, its novelty lies in the formal integration of existing trust mechanisms through explicitly defined interaction constraints that govern cross-layer trust propagation.

The resulting trust pipeline connects TEE-enabled sensing, Byzantine-aware environmental state aggregation, and blockchain-based state commitment to achieve consistent environmental-state consensus in adversarial IoT sensing environments.

## 2. Preliminaries

In this section, we establish the fundamental concepts and notations underpinning the proposed blockchain-enabled environmental-state consensus framework. The motivating application context arises from smart agriculture and AIoT-enabled edge environments, where distributed sensor observations must be reliably aggregated, verified, and coordinated under resource constraints and potential data corruption or adversarial conditions.

### 2.1. Background

Recent advances in smart agriculture have increasingly integrated Internet of Things (IoT), edge computing, and data-driven sensing technologies to improve resource efficiency, environmental monitoring, and agricultural sustainability [3,4,5,33,34]. Precision agriculture systems commonly rely on distributed sensors, embedded devices, and intelligent computing infrastructures for monitoring environmental variables such as temperature, humidity, soil moisture, light intensity, and CO_2_ concentration [9,10,35,36]. These systems increasingly operate under AIoT and edge computing paradigms, where sensing, communication, and local processing are performed close to the physical environment rather than relying solely on centralized cloud infrastructures [14,37,38].

However, real-world agricultural deployments are characterized by heterogeneous sensing devices, resource-constrained embedded systems, intermittent connectivity, and unreliable or partially compromised off-chain data sources. While machine learning and AI-based modules may be used for auxiliary prediction or automation tasks [11,12,13,15,16], the focus of this work is not on AI model design, but on the trustworthy aggregation and consistent coordination of distributed sensing data in edge-enabled environments. Existing cloud-centric approaches often suffer from latency, communication overhead, and energy inefficiency [39,40,41,42], while high-performance computing solutions remain impractical in many rural deployment scenarios due to cost and power constraints [43,44]. These limitations motivate the need for a secure and lightweight coordination framework for distributed sensing systems.

### 2.2. Block Machine

Within this context, we define $B=(B_{1},B_{2},\ldots ,B_{k})$ as a blockchain represented as an ordered sequence of blocks. Each block is formally defined as

(1) $$B_{i}=\left(D_{i},h_{i-1},\sigma_{i}\right),$$

where $D_{i}$ denotes the on-chain data payload, $h_{i-1}$ represents the cryptographic hash of the previous block, and $\sigma_{i}$ denotes the digital signature of the block producer. This chained structure provides tamper-evident linkage between successive blocks and ensures historical consistency of recorded data.

In practical smart agriculture and IoT deployments, a significant portion of sensor observations, environmental measurements, and edge computation outputs cannot be stored entirely on-chain due to scalability and latency constraints. We therefore distinguish between on-chain data $D_{i}$ and off-chain data objects $D_{i}^{\mathrm{off}}$, which may include sensor readings, greenhouse monitoring records, and edge-generated intermediate states. To preserve integrity, off-chain data are associated with on-chain cryptographic commitments as $h_{\mathrm{on}\text{-}\mathrm{chain}}=h\left(D_{i}^{\mathrm{off}}\right)$. This mechanism allows verification of whether off-chain data have been modified after commitment, where tampered data are denoted as $D_{i}^{\mathrm{off}*}$.

We define a lightweight consistency checking abstraction as $\mathrm{Detect}(D_{i}^{\mathrm{off}},h_{\mathrm{on}\text{-}\mathrm{chain}})$, which outputs 1 if the off-chain data are consistent with the on-chain commitment and 0 otherwise. This abstraction models integrity verification between off-chain sensing data and blockchain-recorded commitments in distributed agricultural systems, without assuming correctness of the underlying physical measurements.

The cryptographic hash function h(·) is assumed to be collision-resistant, ensuring that it is computationally infeasible for any probabilistic polynomial-time (PPT) adversary to construct two distinct inputs with the same hash output except with negligible probability. The hash $h(B_{i})$ thus serves as a compact commitment to block contents and historical state.

Importantly, we distinguish between data integrity and physical correctness. Cryptographic mechanisms such as hash commitments and Trusted Execution Environments (TEEs [17,18,19]) ensure that data have not been altered after capture or during trusted processing, but they do not guarantee that sensor readings accurately reflect the true physical environment. TEEs are therefore modeled as isolated execution environments that provide hardware-enforced protection for off-chain data preprocessing and verification tasks in edge gateways and embedded devices.

From a computational perspective, we use the classical Turing machine abstraction M as a general model for computable functions. A function *f* is computable if there exists a Turing machine $M_{f}$ that computes it. Smart contracts executed within Blockchain Virtual Machines (BVMs [45,46,47]) are interpreted as programs running under resource constraints such as bounded execution steps and gas limits, providing a controlled execution environment for decentralized computation.

Security adversaries are modeled as probabilistic polynomial-time (PPT) algorithms A parameterized by a security parameter λ. We use negl(λ) to denote a negligible function. We further define α as the adversarial fraction of consensus resources, including computational power in Proof-of-Work systems or staking power in Proof-of-Stake systems. The system is designed to remain robust under bounded adversarial influence, where α does not dominate the honest majority assumption. The principal notation used throughout this paper is summarized in Table 1.

### 2.3. System Model and Trust Architecture

We model the proposed system as a 4-layer trust architecture, where the transition of domain states is governed by strict security invariants, including

1. **Sensing Layer:** A set of distributed agricultural sensors $S=\{s_{1},\ldots ,s_{n}\}$ producing raw observations that may be noisy or adversarial.
2. **TEE-Assisted Edge Layer:** Each edge node $e_{i}$ is equipped with a Trusted Execution Environment that serves as a hardware-rooted trust anchor, providing attested preprocessing and ensuring that only verifiable computations contribute to downstream aggregation.
3. **Byzantine Aggregation Layer:** A subset of edge outputs are aggregated using a Byzantine-robust mechanism that filters out corrupted inputs up to an α fraction.
4. **Blockchain Coordination Layer:** The final environmental state is recorded on-chain as a tamper-evident and globally consistent reference state.

We define the environmental-state consensus function as

(2) $$F:D_{sensor}\to S_{env}$$

where $D_{sensor}$ denotes distributed sensor observations and $S_{env}$ denotes a consistent environmental-state representation.

To ensure trustworthy environmental-state commitment, a state $S_{t}$ is accepted by the blockchain coordination layer only if it satisfies both execution-level authentication and sensing-level consistency requirements. Specifically, the committed state is defined as:

(3) $$S_{t}\in S_{\mathrm{auth}}\cap S_{\mathrm{Byz}}$$

where $S_{\mathrm{auth}}$ denotes the set of sensing outputs generated under verified execution integrity that

(4) $$S_{\mathrm{auth}}=\left\{x_{i}\left(t\right)|\mathrm{execution}\;\mathrm{integrity}\;\mathrm{verified}\right\}$$

The set $S_{\mathrm{auth}}$ does not imply that the corresponding sensing measurements are physically correct. Instead, it indicates that the sensing outputs are produced through a protected execution path and have not been modified during trusted execution.

The set $S_{\mathrm{Byz}}$ represents sensing states that remain consistent after Byzantine-resilient filtering that

(5) $$S_{\mathrm{Byz}}=\left\{x_{i}\left(t\right)|\mathrm{consistent}\;\mathrm{under}\;\mathrm{Byzantine}\;\mathrm{filtering}\right\}$$

Therefore, the final environmental state committed to the blockchain is generated only from sensing outputs that are both execution-authenticated and Byzantine-consistent. This design introduces an explicit cross-layer trust dependency: the TEE provides an execution-trust boundary, Byzantine-resilient aggregation provides sensing reliability, and blockchain commitment provides state integrity.

Unlike conventional IoT or blockchain-based sensing architectures that directly record sensor measurements, the proposed framework does not assume that trusted execution alone guarantees sensing correctness. Instead, TEE-protected outputs are treated as trusted inputs for subsequent adversarial-aware aggregation, after which only consensus-consistent environmental states are committed to the blockchain.

In this work, the TEE therefore functions as a deployed trust anchor for execution integrity rather than a mechanism for validating physical sensor truth. The reliability of environmental-state estimation is achieved through the combination of TEE-enabled sensing, Byzantine-resilient aggregation, and blockchain-based state commitment.

### 2.4. System Positioning

Recent research has explored several directions that are closely related to this work. Trusted Execution Environment (TEE) and blockchain integration has been widely studied for secure data sharing and trusted execution in IoT systems [22,29,48]. These approaches primarily focus on ensuring execution integrity and tamper-evident storage of data once it enters the system, assuming that sensed data are sufficiently reliable.

In parallel, Byzantine fault-tolerant consensus mechanisms have been enhanced using hardware-assisted techniques and TEE support to improve scalability and robustness of distributed agreement [20,23,24,28]. However, these methods mainly operate at the consensus layer and do not explicitly model the reliability of physical sensing data.

Furthermore, blockchain-enabled IoT and cyber–physical systems have been proposed for secure coordination in domains such as supply chains and energy systems [21,26,27]. While these systems improve data integrity after submission, they typically assume that sensor-generated inputs are already trustworthy and semantically valid.

More recent end-to-end Trusted Execution Environment (TEE)-based system designs aim to extend trust guarantees across distributed IoT infrastructures by unifying secure execution, communication, and data processing pipelines within heterogeneous environments [29,31,32]. While these approaches improve system-wide execution integrity and provide end-to-end protection of computation and data handling workflows, they largely assume that inputs entering trusted environments are inherently valid or sufficiently reliable. Consequently, they do not explicitly model or enforce the separation between execution trust, sensing-layer uncertainty, and state-level consensus under adversarial conditions.

In contrast to these prior works, the key novelty of this paper lies in identifying and formalizing a missing abstraction: a trust-conditioned environmental-state consensus mechanism for adversarial IoT sensing systems. Instead of treating Trusted Execution Environments, Byzantine aggregation, and blockchain as independent or loosely coupled components, we explicitly define their interaction constraints and dependency relationships in a unified system model.

To the best of our knowledge, existing approaches either (i) guarantee execution integrity without validating sensing reliability, (ii) provide Byzantine robustness without distinguishing trusted execution contexts, or (iii) ensure ledger immutability without addressing pre-commitment data correctness. As summarized in Table 2, while contemporary solutions often address specific facets of decentralized trust in isolation, our framework integrates these functionalities into a unified pipeline. TEE-accelerated BFT systems focus on computation and aggregation but lack state consensus guarantees. TEE-blockchain and Byzantine consensus systems prioritize either ledger immutability or robust aggregation, yet fail to provide comprehensive end-to-end reliability. Furthermore, existing end-to-end TEE solutions often operate outside the decentralized ledger paradigm.

In contrast, the proposed system is the only approach that concurrently satisfies TEE execution, Byzantine-resilient aggregation, blockchain-based storage, and formal state consensus. By introducing a cross-layer trust pipeline that jointly governs execution, aggregation, and state commitment, our framework effectively bridges these gaps. Consequently, this holistic design ensures robust performance and data integrity, achieving a reliable trust model that remains resilient even under adversarial sensing conditions.

## 3. Secure Blockchain Consensus Computing

Based on the information-theoretic and adversarial models introduced in the previous sections, we present a blockchain-enabled trusted computing architecture for agricultural AIoT systems. The proposed framework integrates a Blockchain Virtual Machine (BVM), a Trusted Execution Environment (TEE)-enabled edge platform, and an on-chain/off-chain integrity preservation mechanism. The objective of this architecture is not to provide a new blockchain consensus protocol, but rather to ensure trustworthy environmental state commitment under adversarial sensing conditions.

### 3.1. Threat Model and Security Assumptions

The proposed blockchain-enabled agricultural AIoT framework operates under a partially trusted execution model consisting of distributed sensing nodes, edge gateways, blockchain validators, and a Trusted Execution Environment (TEE) infrastructure.

The security analysis is based on the following assumptions.

**Assumption** **1** (A1 Trusted Execution Infrastructure)**.** *The underlying TrustZone/OP-TEE infrastructure provided by the edge platform correctly enforces execution isolation, secure memory protection, and cryptographic key confidentiality. The TEE is assumed to execute deployed aggregation and blockchain commitment logic faithfully.*

**Assumption** **2** (A2 Compromised Sensing Nodes)**.** *A subset of sensing nodes may be compromised by an adversary and can generate arbitrary falsified environmental observations.*

**Assumption** **3** (A3 Untrusted Sensor Inputs)**.** *Although the TEE provides execution integrity, it does not guarantee that sensor observations faithfully represent the underlying physical environment. Consequently, corrupted measurements may still enter the trusted execution pipeline.*

**Assumption** **4** (A4 Permissioned Validators)**.** *Blockchain validators are operated under a permissioned authority-based management model and are assumed to follow protocol rules unless explicitly stated otherwise.*

Under these assumptions, the adversary is allowed to (1) compromise sensing nodes; (2) inject arbitrary environmental observations; (3) coordinate multiple compromised nodes; (4) attempt to influence blockchain commitment decisions through manipulated sensing data. However, the adversary is assumed incapable of compromising the underlying TrustZone/OP-TEE trusted execution infrastructure.

### 3.2. Security Objectives and Scope

The primary objective of this work is not to establish a new blockchain consensus protocol from first principles. Instead, the focus is on preserving sensing-state integrity in adversarial agricultural environments where sensing nodes may become compromised. Accordingly, the proposed framework pursues the following security objectives.

**Objective** **1** (SO1: Environmental State Integrity)**.** *Environmental states committed into the blockchain should remain consistent with the underlying physical process despite the presence of compromised sensing nodes.*

**Objective** **2** (SO2: Resilience Against Adversarial Sensing Attacks)**.** *The aggregation mechanism should suppress manipulated observations and reduce the influence of Byzantine sensing behaviors.*

**Objective** **3** (SO3: Trusted Execution of Aggregation Logic)**.** *Aggregation, verification, and blockchain commitment procedures should execute within a trusted environment that protects computation integrity and cryptographic assets.*

The scope of this work is limited to sensing-layer adversarial resilience and trusted execution of aggregation procedures. The manuscript does not attempt to formally prove the security of the underlying blockchain consensus protocol itself.

### 3.3. System Architecture

**Figure 1:**
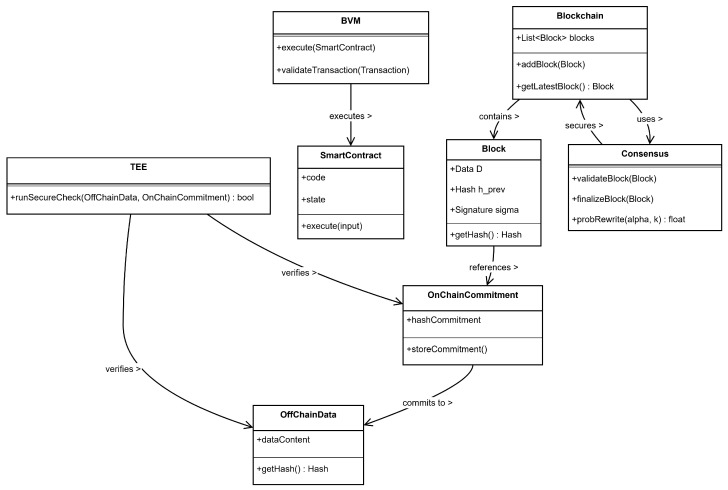
Blockchain Virtual Machine and TEE-Enabled Edge Architecture.

Figure 1 illustrates the overall architecture of the proposed framework.

The Blockchain maintains an ordered sequence of blocks containing environmental state commitments, transaction records, and references to external data objects. The Blockchain Virtual Machine (BVM) executes smart contract logic associated with sensing, verification, and environmental state management.

Although the BVM supports theoretically Turing-complete computation, practical execution is constrained by resource limits such as gas consumption, execution time, and memory availability. Therefore, all security discussions in this work refer to bounded executable smart contract programs rather than unrestricted Turing-complete computation.

Large sensing datasets and historical environmental records are maintained in off-chain storage systems due to scalability and storage efficiency considerations. Integrity protection is achieved through cryptographic commitments anchored on the blockchain.

The trusted execution layer is implemented through a TrustZone/OP-TEE-enabled edge platform provided by an industrial partner. The TEE infrastructure is treated as a trusted execution substrate responsible for protecting aggregation logic, cryptographic operations, and blockchain commitment procedures against software-level tampering.

Importantly, the TEE does not guarantee the correctness of sensing observations. Compromised sensing devices may still inject manipulated measurements into the sensing pipeline. Consequently, adversarial robustness is primarily achieved through Byzantine-resilient aggregation and validation mechanisms rather than through the TEE itself.

The Consensus module is responsible for determining how environmental observations are filtered, aggregated, and committed into the blockchain ledger. Different aggregation strategies can be deployed within the same trusted execution environment without modifying the underlying TEE infrastructure.

This layered architecture separates execution integrity from sensing integrity, allowing trusted execution and adversarially robust consensus mechanisms to be analyzed independently.

#### TEE Deployment Scope and Trust Boundary

In the proposed architecture, the TEE component is deployed at the field sensing layer rather than within the blockchain coordination layer. The sensing devices employ STM32L5-series microcontrollers based on the ARM Cortex-M33 TrustZone architecture, where secure execution and device-side data protection are provided by the underlying trusted firmware environment.

The proposed framework does not modify the internal TEE implementation or control its lifecycle operations, including activation, revocation, or secure-world configuration. Instead, the blockchain layer receives authenticated outputs generated by the TEE-enabled sensing devices and utilizes these outputs as trusted inputs for Byzantine-resilient aggregation and ledger commitment.

Therefore, the TEE serves as a trust boundary between physical sensing and distributed coordination, reducing the influence of compromised sensing nodes while preserving compatibility with existing hardware security infrastructures.

### 3.4. Consensus Validation Framework

**Figure 2:**
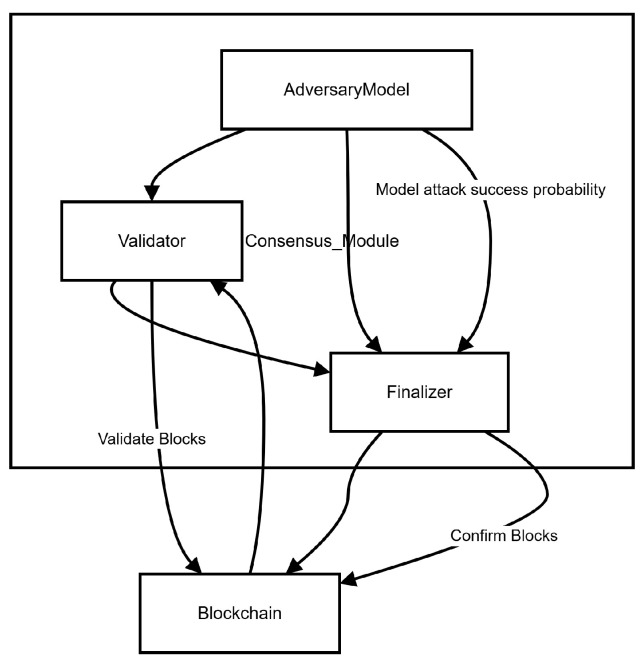
Component Interactions of the Consensus Validation Framework.

Figure 2 illustrates the interaction between blockchain validation, environmental state aggregation, and final commitment procedures. The Validator component verifies transaction authenticity, cryptographic signatures, and environmental state consistency prior to blockchain commitment. The Finalizer determines whether sufficient evidence exists to accept an environmental state update.

Rather than modeling a specific Proof-of-Work or Proof-of-Stake protocol, the proposed framework adopts a permissioned environmental-state ledger architecture. Finality is therefore determined by validator agreement and confirmation policies rather than by Nakamoto-style probabilistic chain growth assumptions.

The adversarial model considered in this work focuses primarily on sensing-layer attacks, including compromised sensing nodes, falsified environmental observations, and collusive manipulation attempts. The objective is to preserve the integrity of committed environmental states despite adversarial sensing behaviors. Consequently, the consensus evaluation emphasizes sensing-state robustness and environmental consistency rather than low-level blockchain consensus security proofs.

### 3.5. Off-Chain Integrity Interaction Protocol

**Figure 3:**
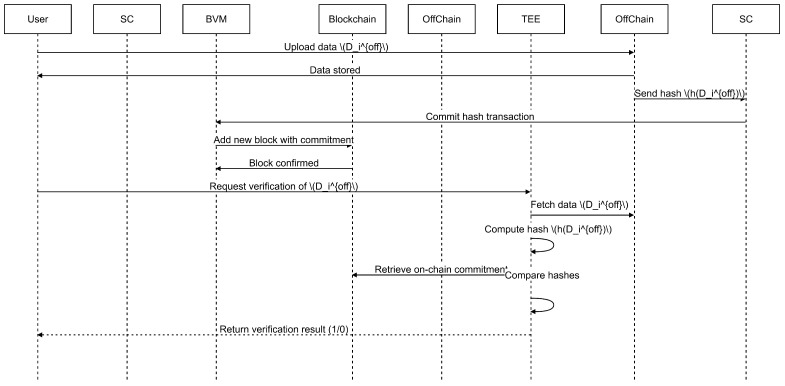
Interaction protocol phases. Note: Duplicate entities (SC, OffChain) denote different functional interfaces or distinct operational stages (data submission vs. data retrieval) within the protocol architecture.

Figure 3 illustrates the interaction process between off-chain storage, blockchain commitments, and trusted execution components. Environmental datasets may be stored in distributed storage systems such as IPFS or external databases. When new data are generated, a cryptographic hash commitment is computed and anchored on the blockchain through a smart contract transaction.

As shown in the figure, the protocol separates the lifecycle into two main stages, including the Data Commit Phase and the Integrity Verification Phase. To maintain clarity regarding the distinct operational interfaces, we have represented the Smart Contract (SC) and Off-chain storage (OffChain) entities at both their data-submission and verification entry points. Subsequent integrity verification involves retrieving the stored data and recomputing the associated cryptographic commitment. Verification logic is executed inside the trusted execution environment to protect cryptographic operations and commitment verification procedures from software tampering.

A successful verification confirms consistency between the off-chain data object and its corresponding blockchain commitment. This mechanism provides integrity assurance without requiring large environmental datasets to be stored directly on-chain. The protocol therefore combines blockchain immutability, cryptographic commitments, and trusted execution to support scalable environmental data management.

### 3.6. BVM Execution Lifecycle

**Figure 4:**
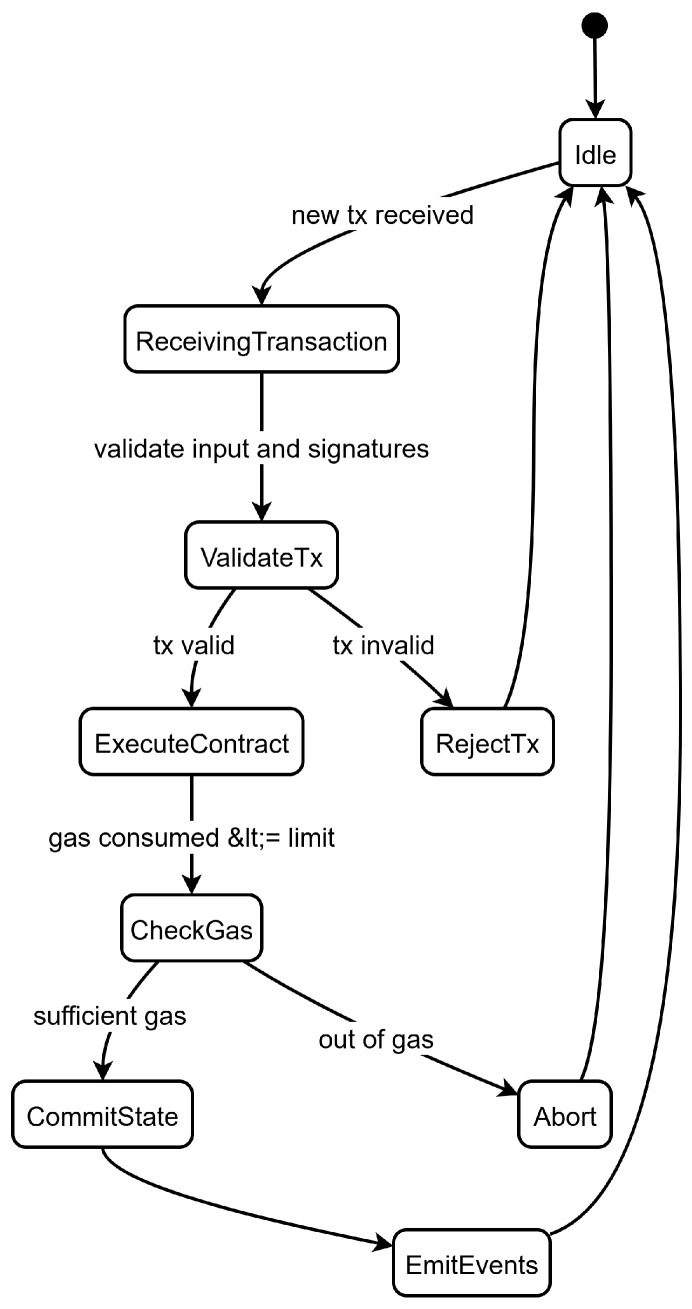
Workflow of the BVM execution lifecycle.

Figure 4 illustrates the execution lifecycle of a smart contract within the Blockchain Virtual Machine. Upon receiving a transaction request, the BVM performs signature verification, input validation, and authorization checks. Invalid transactions are rejected before execution.

For valid transactions, contract logic is executed under predefined resource constraints, including execution-time limits and gas consumption policies. These restrictions ensure deterministic execution and prevent denial-of-service behaviors associated with unrestricted computation. If execution completes successfully within the permitted resource budget, the resulting state transition is committed to the blockchain ledger. Otherwise, execution is aborted and state changes are discarded.

This bounded execution model provides predictable behavior, facilitates formal reasoning about smart contract execution, and aligns practical deployment constraints with the theoretical expressiveness of the BVM.

## 4. On–off-Chain Interaction Protocol

**Figure 5:**
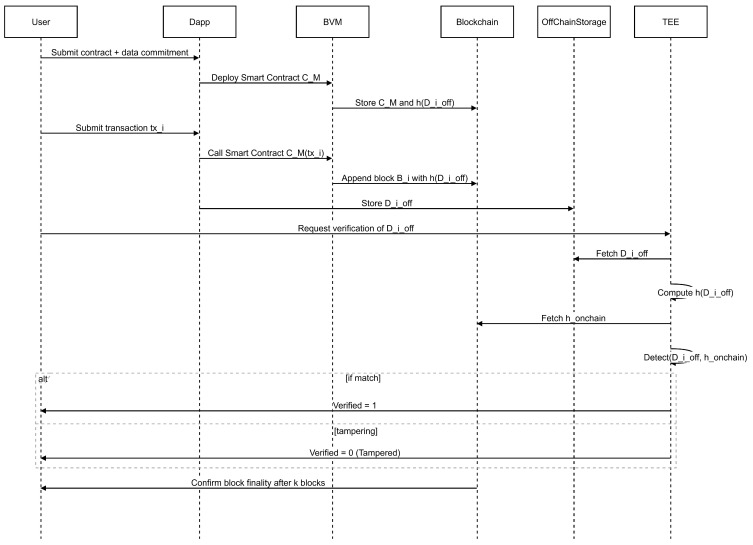
On–Off-Chain Protocol.

The on–off-chain interaction protocol defines a cryptographically verifiable mechanism for binding off-chain data with blockchain state transitions in a permissioned AIoT environment. Figure 5 illustrates the interaction workflow, including smart contract execution within the Blockchain Virtual Machine (BVM), on-chain commitment of data hashes, and off-chain integrity verification using a Trusted Execution Environment (TEE)-enabled infrastructure.

### 4.1. Interaction Workflow

The protocol begins with a user submitting a smart contract and an off-chain data object $D_{i}^{\mathrm{off}}$ to a decentralised application (DApp). The smart contract C is deployed onto the Blockchain Virtual Machine (BVM), and a cryptographic commitment $h(D_{i}^{\mathrm{off}})$ is recorded on-chain. When a transaction $tx_{i}$ is submitted, it is executed by the BVM under deterministic and resource-bounded execution rules. The resulting state transition is appended to a new block $B_{i}$, which contains the hash commitment and state update.

Off-chain data $D_{i}^{\mathrm{off}}$ is stored in an external repository due to scalability constraints. For integrity verification, a Trusted Execution Environment (TEE) provided by an external industrial platform is used to execute verification logic in a protected environment. The TEE recomputes the hash of retrieved data and compares it with the on-chain commitment.

Importantly, the TEE is treated as a trusted execution substrate and is not responsible for validating the correctness of sensing or data generation processes. Its role is limited to protecting cryptographic verification operations from software-level tampering. Finality is achieved after *k* subsequent blocks are appended under the permissioned consensus protocol.

### 4.2. Contract Deployment and Data Commitment

In the proposed system, smart contracts are used as deterministic state-management programs within the permissioned blockchain layer. Their primary function is to record validated environmental-state commitments generated from distributed sensing and edge processing, rather than to execute complex on-chain computations.

To avoid storing large volumes of sensor data on-chain, the system adopts a hash-based commitment mechanism. Let $D_{i}^{\mathrm{off}}$ denote an environmental-state record stored off-chain. A collision-resistant hash function h(·) is used to generate an immutable commitment as

(6) $$h_{\mathrm{on}\text{-}\mathrm{chain}}=h\left(D_{i}^{\mathrm{off}}\right).$$

The resulting hash value is recorded on the blockchain together with the corresponding state metadata. This mechanism enables integrity verification and traceability while maintaining low storage overhead on resource-constrained IoT infrastructures.

Whenever an environmental-state record is queried or audited, the off-chain data can be rehashed and compared with the committed on-chain value. Any modification of the original record results in a hash mismatch, thereby providing tamper-evident verification of sensing outcomes and state-construction results.

Within the proposed framework, blockchain commitments serve as immutable records of validated environmental states. Only states that have passed TEE-assisted processing and Byzantine-resilient aggregation are eligible for commitment, establishing a direct connection between sensing reliability and ledger integrity.

### 4.3. Transaction Execution and State Transition

Each transaction is executed deterministically by the BVM under a globally replicated state model. A block is defined as

(7) $$B_{i}=\left(D_{i},h_{i-1},\sigma_{i}\right),$$

where $D_{i}$ represents the state update, $h_{i-1}$ is the previous block hash, and $\sigma_{i}$ is a digital signature. The system assumes standard cryptographic primitives such as collision-resistant hash functions and EUF-CMA-secure signatures to ensure integrity and authenticity under standard adversarial assumptions. To ensure system liveness and prevent resource exhaustion, execution is constrained by gas limits and computational budgets.

### 4.4. Consensus and Finality Guarantees

The system operates under a permissioned consensus model inspired by Proof-of-Authority (PoA). Finality is determined by validator quorum agreement and confirmation depth *k*, rather than probabilistic mining models such as Proof-of-Work or Proof-of-Stake. The probability expression can be formulated as

(8) $$P={\left(\frac{\alpha }{1-\alpha }\right)}^{k}$$

which is used only as an illustrative model to capture the qualitative effect of confirmation depth under adversarial participation. It is not intended to represent the exact security bound of the deployed permissioned ledger. In this setting, α is interpreted as an abstract adversarial influence factor within the sensing and validation process, rather than a strict computational power ratio.

### 4.5. Security Considerations

The security of the proposed protocol relies on standard cryptographic assumptions, including collision resistance of hash functions and authenticity of digital signatures. The Trusted Execution Environment (TEE) is assumed to provide execution isolation for verification logic, based on an external industrial deployment. The TEE is treated as a fixed infrastructure component and is not the subject of implementation or performance evaluation in this work. Security analysis focuses on data integrity and state consistency under adversarial sensing conditions, rather than full consensus protocol verification.

## 5. Blockchain-Driven Trusted Execution

### 5.1. Blockchain Virtual Machine and Computational Model

At the core of the proposed architecture lies a Blockchain Virtual Machine (BVM), which serves as an abstract execution layer for smart contract deployment and decentralized computation. The BVM is designed to support general-purpose programmable logic for IoT coordination and data-driven decision-making. In principle, smart contracts deployed on the BVM can encode arbitrary deterministic computation subject to resource-bounded execution constraints.

To ensure practical executability, the system enforces bounded execution semantics via resource control mechanisms such as gas metering or instruction limits. These constraints prevent non-termination and mitigate denial-of-service behaviors, while preserving the expressiveness required for decentralized applications.

### 5.2. Immutable Ledger and Cryptographic Commitment

The blockchain ledger is modeled as an ordered sequence of blocks $B=(B_{1},B_{2},\ldots ,B_{k})$, where each block is defined as (7). Assuming that the underlying hash function h(·) is collision-resistant and the signature scheme is existentially unforgeable under chosen-message attacks (EUF-CMA), the ledger ensures tamper-evident integrity. Any modification of historical data would require recomputing a valid cryptographic chain, which is computationally infeasible under standard cryptographic assumptions.

To support scalable IoT data processing, the system employs an off-chain data storage mechanism with on-chain cryptographic commitments. Each off-chain data object $D_{i}^{\mathrm{off}}$ is associated with a commitment defined by (6) which is recorded on-chain to bind the external data to the immutable ledger. This design allows large-scale or privacy-sensitive data to remain off-chain while preserving verifiability through hash-based authentication. Integrity verification is performed by recomputing and comparing cryptographic digests when data is retrieved for processing or auditing.

### 5.3. Trusted Execution Environment as Fixed System Assumption

The proposed architecture assumes the availability of a Trusted Execution Environment (TEE) at selected edge nodes. The TEE is provided by the underlying deployment infrastructure (e.g., ARM TrustZone/OP-TEE-enabled gateways) and is used as a *fixed trusted execution substrate*.

Importantly, the TEE is not treated as a tunable or evaluated component in this work. Instead, it remains identical across all experimental configurations and does not participate in the ablation study. Therefore, all performance differences reported in this paper are not attributed to variations in TEE implementation or availability, but to differences in consensus-layer and aggregation-layer design.

Within this assumed trusted environment, integrity verification of off-chain data can be executed securely. Specifically, when a data object $D_{i}^{\mathrm{off}}$ is accessed, the enclave computes $h(D_{i}^{\mathrm{off}})$ and compares it with the on-chain commitment $h_{\mathrm{on}-\mathrm{chain}}$:

(9) $$\mathrm{Verify}\left(D_{i}^{\mathrm{off}},h_{\mathrm{on}-\mathrm{chain}}\right)=\left\{\begin{array}{cc} 1, & \mathrm{if}\;h\left(D_{i}^{\mathrm{off}}\right)=h_{\mathrm{on}-\mathrm{chain}},\\ 0, & \mathrm{otherwise}. \end{array}\right.$$

This verification procedure is executed within the trusted hardware boundary. However, in our experimental design, it remains a constant system primitive rather than a variable under evaluation.

### 5.4. Logic Decomposition and Ablation Scope

To address concerns regarding attribution of performance improvements, we explicitly decompose the system into three conceptual layers, including

1. **Trusted Execution Layer**, which provides secure execution via TEE. This layer is fixed and identical across all experimental settings and is not part of the ablation space.
2. **Aggregation Layer**, which defines how multi-source IoT updates are aggregated, including Vanilla (no robust filtering), statistical aggregation (mean-based), and Byzantine-resilient aggregation mechanisms.
3. **Confirmation Layer** with *K*-confirmation logic for transaction finality under probabilistic consensus guarantees.

Under this decomposition, the ablation study focuses exclusively on variations within the aggregation and confirmation layers while holding the trusted execution environment constant.

### 5.5. Consensus Robustness and Probabilistic Finality

To quantify security against chain reorganization attacks, we adopt a probabilistic model based on adversarial control fraction α. The probability that an adversary can successfully rewrite a block after *k* confirmations is bounded by

(10) $$P_{\mathrm{rewrite}}\leq {\left(\frac{\alpha }{1-\alpha }\right)}^{k},$$

for α<0.5. This expression captures the exponential decay of attack success probability as confirmation depth increases, providing a formal justification for *K*-confirmation-based finality.

### 5.6. Workflow Integration

The overall system workflow integrates blockchain execution, off-chain data commitment, and trusted verification under a unified architecture. IoT devices generate data that is stored off-chain and committed on-chain via cryptographic hashes. Aggregation and consensus mechanisms operate over these committed values to ensure consistency and fault tolerance. When verification is required, TEE-based execution is invoked under the assumption of a trusted substrate, ensuring integrity validation without exposing sensitive computation to the untrusted environment.

It is important to emphasize that the Trusted Execution Environment is not a variable in the experimental evaluation. All reported performance differences arise from the design of aggregation and confirmation mechanisms under identical trusted execution conditions. This ensures that the ablation results isolate the contribution of the proposed consensus-layer improvements rather than differences in underlying hardware security infrastructure.

## 6. Experimental System in Smart Agriculture

### 6.1. System Perspective

**Figure 6:**
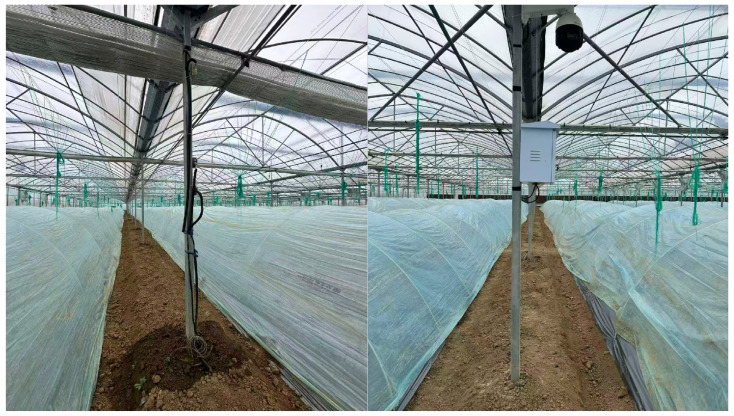
Farm System on Site.

The proposed experimental system is designed as a blockchain-enabled IoT infrastructure for smart agriculture, where distributed sensing, edge intelligence, wireless communication, and consensus-driven state validation are tightly integrated into a unified system. Figure 6 shows the system deployed in field. This greenhouse IoT system serves as a physical instantiation of the general environmental-state consensus problem.

In this system, rather than treating blockchain as an isolated transactional subsystem, we reinterpret it as a consensus abstraction layer over distributed environmental sensing. In this architecture, environmental observations generated by IoT devices are not treated as financial transactions but as state-transition evidence for physical-world estimation. Consequently, the blockchain layer serves as an integrity-preserving coordination mechanism that ensures tamper-resistant environmental state finalisation under noisy and adversarial conditions.

### 6.2. IoT System Architecture

**Figure 7:**
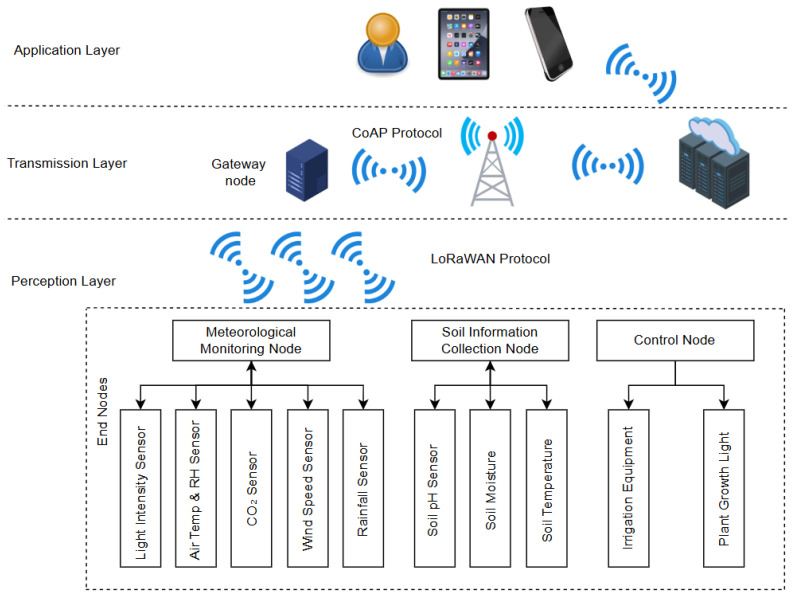
Three-Layer IoT System Architecture for Smart Agriculture.

The system adopts a layered IoT architecture for scalable agricultural deployment. As shown in Figure 7, the architecture consists of perception, transmission, and application layers. The perception layer contains heterogeneous sensors and actuators measuring temperature, humidity, soil moisture, CO_2_, light intensity, and irrigation status. The transmission layer provides low-power communication using LoRa, WiFi, and NB-IoT. The application layer provides visualisation, monitoring, and computing interfaces for agricultural decision-making.

To ensure end-to-end data integrity and prevent unauthorized control commands, a Trusted Execution Environment (TEE) is integrated into the transmission layer. Specifically, the gateway node (implemented via ARM TrustZone technology) serves as the primary deployment anchor for the TEE. Since the transmission layer acts as a critical bridge responsible for protocol translation, converting low-power perception protocols like LoRaWAN to application-layer protocols like CoAP/MQTT, which is highly vulnerable to physical tampering and credential theft. By isolating cryptographic keys, decryption routines, and edge computing algorithms within the secure world of the TEE, the architecture guarantees that data transit and command validation remain resilient against both software exploits and hardware-level attacks at the edge.

**Figure 8:**
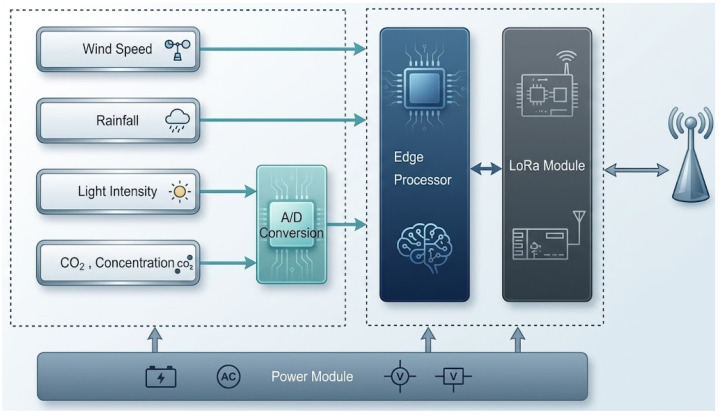
IoT-Based Sensing, Edge Processing, and Platform Architecture.

Figure 8 further details the system pipeline, where sensor data flows through MQTT-based communication into system platforms composed of databases, big-data APIs, and decision services. This enables a closed-loop computing system for irrigation, lighting, and environmental regulation.

### 6.3. Physical System

The system adopts a heterogeneous edge architecture consisting of resource-constrained sensing devices, TEE-enabled sensing platforms, edge gateways, and blockchain coordination nodes. Sensing devices are responsible for environmental data acquisition and transmission. These devices may operate in partially trusted environments and therefore are not assumed to provide complete hardware-level security guarantees. The collected measurements are treated as potentially noisy or adversarial observations rather than directly trusted environmental states.

To establish a trusted boundary between physical sensing and distributed coordination, selected sensing platforms employ Trusted Execution Environment (TEE) support based on ARM TrustZone-M enabled microcontrollers. Specifically, STM32L5-series devices with ARM Cortex-M33 TrustZone architecture are adopted as field-level trusted sensing components. The secure execution environment is provided by the deployed device firmware and is responsible for protecting selected sensing-side operations and generating authenticated data outputs.

The proposed blockchain framework does not modify the internal TEE implementation or control its lifecycle, including secure-world activation, revocation, or configuration. Instead, the blockchain coordination layer receives authenticated sensing outputs produced by the TEE-enabled sensing platform and performs subsequent Byzantine-resilient state aggregation, consensus coordination, and blockchain-based state commitment.

Therefore, TEE is not introduced as a newly designed hardware security mechanism in this work. Instead, it serves as a deployed trusted execution anchor that provides integrity-protected sensing outputs and reduces the risk of unverified data entering the distributed coordination process.

The TEE component is used to establish a trusted execution boundary for sensing-related operations and cryptographic protection, while the correctness of physical observations is further addressed through Byzantine-resilient aggregation mechanisms.

Although TEE-enabled devices provide authenticated execution and protected data handling, the physical sensing process remains affected by measurement uncertainty and environmental disturbances. Let the underlying physical process be denoted as x(t). The sensor output can be expressed as the noisy observations generated by each sensor node as

(11) $$x_{i}\left(t\right)=x\left(t\right)+\epsilon_{i}\left(t\right),\;\epsilon_{i}\left(t\right)∼N\left(0,\sigma^{2}\right),$$

representing stochastic measurement noise.

To model adversarial cyber–physical threats, each sensor includes an attack activation mechanism. When enabled, the observation becomes

(12) $$x_{i}^{attack}\left(t\right)=x\left(t\right)+\delta_{i}\left(t\right),\;\delta_{i}\left(t\right)∼U\left(-5,5\right).$$

This abstraction represents cyber–physical sensing attacks, including spoofing, replay-based manipulation, signal injection, and sensor calibration deviations. Such attacks occur at the sensing layer and cannot be fully prevented by the TEE alone, motivating the use of Byzantine-resilient aggregation. Gateway nodes operate as intermediate coordination entities providing (i) edge data collection, (ii) communication management, (iii) Byzantine-aware aggregation support, (iv) blockchain transaction submission and state commitment.

### 6.4. Edge Consensus and Byzantine-Resilient Aggregation

At the edge layer, a set of sensor observations $V_{t}={\left\{v_{i}\left(t\right)\right\}}_{i=1}^{N}$ is collected at time *t*, where a subset of nodes may behave in a faulty or adversarial manner. A robust aggregation operator is applied to compute a representative environmental estimate

(13) $$x^{*}\left(t\right)=F\left(V_{t}\right),$$

where F(·) denotes a Byzantine-resilient statistical estimator (e.g, mean aggregation).

To capture iterative refinement behavior under distributed processing, we define an abstract update form

(14) $$x^{\left(m+1\right)}\left(t\right)=F\left(x_{1}^{\left(m\right)}\left(t\right),\ldots ,x_{N}^{\left(m\right)}\left(t\right)\right).$$

that describes repeated aggregation under heterogeneous and potentially adversarial inputs, without assuming exact convergence guarantees.

### 6.5. K-Confirmation-Based State Finality

The system adopts a K-confirmation mechanism to regulate when an aggregated environmental state is committed to the ledger. A candidate state at time *t* is considered eligible for commitment if

(15) $$\left|V_{t}\right|\geq K.$$

Once this condition is satisfied, the state may be recorded into the blockchain ledger. Otherwise, the system retains the previous committed state. The finality latency at an abstract level can be fomulated as

(16) $$T_{\mathrm{finality}}∼E\left[T|K,N\right],$$

where *K* controls the confirmation depth and *N* represents system redundancy.

This expression is used to qualitatively describe the trade-off between confirmation strictness and system responsiveness. It is not intended to represent a universal probabilistic bound across different blockchain consensus families.

### 6.6. Blockchain Layer as a Permissioned State Ledger

The blockchain layer is implemented as a lightweight permissioned coordination ledger inspired by Proof-of-Authority (PoA) systems. In this design, the ledger maintains an ordered sequence of environmental state records rather than financial transactions. Each block represents a validated snapshot of the system state at time *t*, including aggregated sensor outputs and integrity commitments.

The role of the ledger is to ensure (1) traceability of historical environmental states, (2) integrity of committed state transitions, (3) ordered recording of system evolution. Consensus in this system is based on validator agreement under a permissioned setting, where validators are assumed to be known entities within the deployment infrastructure. Therefore, finality depends on quorum and policy rules rather than probabilistic mining or staking competition.

The system integrates edge aggregation, K-confirmation, and ledger commitment into a unified environmental state management pipeline. The system adopts a permissioned consortium blockchain jointly deployed with external stakeholders in the smart agriculture infrastructure. Unlike public blockchain systems (e.g., Ethereum), our system does not rely on gas mechanisms or economic fee markets. Instead, block production and state commitment are governed by predefined validator sets and lightweight Byzantine fault-tolerant consensus protocols. Therefore, system performance is evaluated using computational latency, transaction throughput, and commitment delay rather than gas-related metrics.

### 6.7. System-Level Cyber–Physical Control Loop

The system operates as a closed-loop cyber–physical control system. Sensor observations are continuously collected and processed through edge aggregation, producing a sequence of estimated states

(17) $$x^{*}\left(t\right)=F\left(V_{t}\right).$$

These states are then evaluated against predefined control policies. When deviations exceed predefined thresholds, the system triggers actuator responses such as irrigation, ventilation, or lighting control. The closed-loop evolution can be abstracted as

(18) $$x^{*}\left(t\right)\to C\left(x^{*}\left(t\right)\right)\to E_{t+1},$$

where C(·) denotes the control policy and $E_{t+1}$ represents the resulting environmental state.

### 6.8. Experimental Results and System Validation

To evaluate the robustness and operational reliability of the proposed blockchain-enabled agricultural AIoT framework, we conducted a series of adversarial experiments under different aggregation and blockchain coordination strategies while keeping the underlying TEE deployment unchanged. The objective of these experiments is not to benchmark the Trusted Execution Environment itself, but rather to evaluate how authenticated sensing outputs produced by the deployed TEE interact with Byzantine-resilient aggregation and blockchain-based state commitment under different adversarial sensing conditions.

The experiments investigate how adversarial sensing behaviors influence environmental-state consistency, blockchain commitment stability, and consensus reliability when all configurations operate on the same trusted execution infrastructure.

All experimental configurations were executed using the same deployed TrustZone-based trusted execution environment provided by the sensing platform. The underlying TEE implementation, including secure-world execution, key management, and device-side protection, remained unchanged throughout all experiments and therefore was not treated as an experimental variable.

Consequently, the reported results reflect the behaviour of the proposed coordination framework operating on top of an existing trusted execution infrastructure rather than the performance of the TEE implementation itself.

The deployed TrustZone-based Trusted Execution Environment (TEE) serves as the trusted execution substrate of the sensing platform. Its role is to provide authenticated execution for sensing-side preprocessing, aggregation, verification, and blockchain commitment operations while protecting cryptographic materials and execution states from software-level tampering. The underlying TEE implementation, including secure-world execution and device-side protection, is provided by the deployed STM32L5 TrustZone firmware and remains unchanged throughout all experiments. Consequently, the TEE is not treated as an experimental variable, nor is the objective of this work to evaluate or optimise the internal performance of the TrustZone implementation.

Although the TEE provides execution integrity and authenticated data generation, it cannot determine whether the collected sensor observations faithfully represent the underlying physical environment. Sensor faults, spoofing attacks, replay attacks, and malicious data injection may still produce authenticated but incorrect observations. Therefore, trusted execution alone cannot ensure trustworthy environmental-state estimation.

To address this limitation, different aggregation and consensus strategies are evaluated under identical trusted execution conditions. Since every experimental configuration operates on the same deployed TEE infrastructure, any differences in system performance can be attributed to the proposed Byzantine-resilient aggregation and blockchain coordination mechanisms rather than variations in the trusted execution environment. This experimental design isolates the contribution of the proposed coordination framework while maintaining a consistent hardware trust boundary across all experiments.

The physical experimental platform consists of six distributed sensing nodes deployed in the greenhouse environment. During each experiment, Node0 is used as the reference sensing node to provide an approximate ground-truth environmental state for comparison, while the remaining sensing nodes participate in distributed environmental-state consensus. The environmental state ultimately committed to the blockchain is computed from the aggregated observations of these participating nodes according to the selected aggregation strategy.

Accordingly, the overall system can be viewed as a two-layer architecture. The first layer is a trusted execution layer implemented by the deployed TrustZone-based TEE, which provides authenticated sensing outputs and protects aggregation execution, cryptographic operations, and blockchain commitment from software-level attacks. The second layer is the proposed trust-aware coordination layer, which performs Byzantine-resilient aggregation, environmental-state consensus, and blockchain state commitment using the authenticated outputs generated by the TEE.

Based on this architecture, three system configurations are evaluated under the same trusted execution infrastructure, including (1) **Vanilla Mode**, where sensing observations are directly committed without adversarial filtering; (2) **Mean Consensus Mode**, where environmental states are obtained using conventional arithmetic-mean aggregation; (3) **Byzantine Consensus Mode**, where Byzantine-resilient filtering and robust aggregation are performed before blockchain state commitment.

Because all three configurations operate on the same deployed TrustZone environment, the reported results evaluate the effectiveness of the proposed trust-aware coordination framework, including authenticated sensing, Byzantine-resilient aggregation, and blockchain-based environmental-state commitment, rather than the hardware-level performance of the underlying TEE implementation.

#### 6.8.1. Experimental Results Under Vanilla Consensus Mode

The Vanilla mode is evaluated as a baseline without adversarial awareness. Although all experiments are executed on the same TEE-enabled edge platform, this configuration does not activate any integrity verification, anomaly detection, or Byzantine-resilient filtering mechanisms. Therefore, sensor observations are directly aggregated and committed to the blockchain, representing a conventional IoT consensus workflow under trusted-input assumptions.

**Figure 9:**
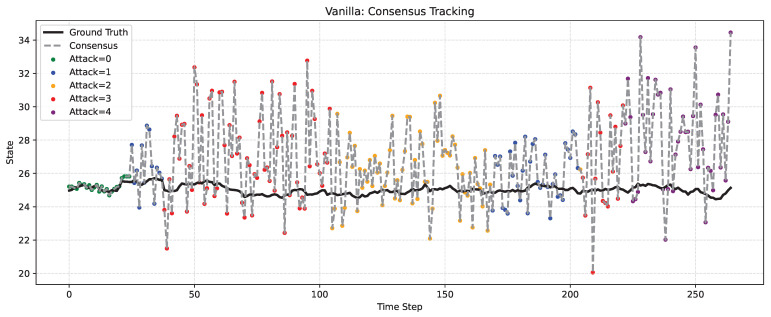
Consensus tracking under Vanilla mode.

**Figure 10:**
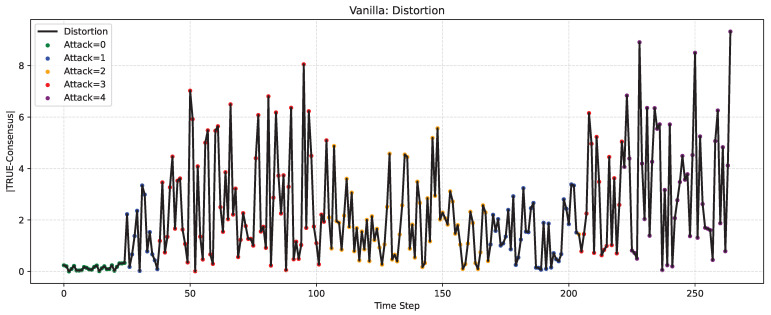
State distortion under Vanilla mode.

As shown in Figure 9 and Figure 10, the Vanilla strategy performs well in benign environments where all sensing nodes provide consistent measurements. The aggregated state closely follows the ground-truth environmental dynamics, and the remaining deviation is mainly caused by sensor noise and communication uncertainty.

However, this behavior rapidly deteriorates once compromised observations are introduced. Because the Vanilla aggregation treats all measurements equally, malicious sensor readings are directly incorporated into the consensus process. Even a small number of compromised nodes can introduce noticeable estimation bias, causing increased state distortion.

As the adversarial ratio increases, corrupted observations gradually dominate the aggregation process, leading to significant divergence between the committed blockchain state and the physical environment. These results indicate that conventional aggregation is insufficient for IoT environments where sensing devices cannot be fully trusted.

#### 6.8.2. Experimental Results Under Mean Consensus Mode

The Mean consensus mode introduces a lightweight statistical improvement by averaging all received sensor observations before blockchain commitment. Unlike the Vanilla strategy, averaging reduces random sensing fluctuations and provides a smoother estimation under normal operating conditions. However, it does not distinguish between trustworthy and adversarial measurements.

**Figure 11:**
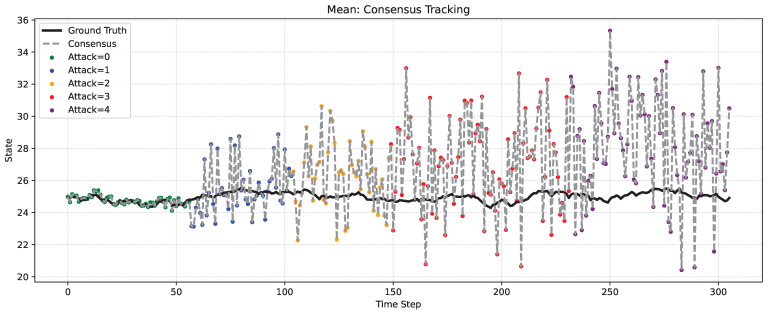
Consensus tracking under Mean aggregation.

**Figure 12:**
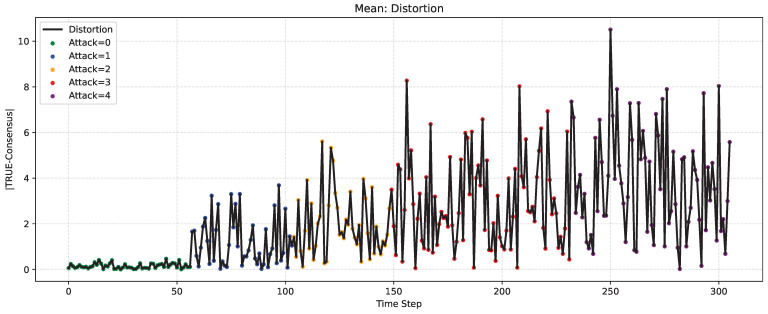
State distortion under Mean aggregation.

As illustrated in Figure 11 and Figure 12, Mean aggregation achieves lower fluctuations than Vanilla under non-adversarial conditions, confirming the benefit of basic statistical smoothing.

Nevertheless, this improvement does not translate into adversarial robustness. Since the arithmetic mean assigns identical importance to all observations, malicious measurements can shift the aggregation result toward an incorrect state. When the number of compromised nodes increases, the influence of corrupted data becomes statistically significant, resulting in increasing bias and distortion.

Compared with Vanilla aggregation, Mean consensus provides better noise tolerance but remains vulnerable to coordinated sensing attacks. Therefore, simple statistical aggregation alone cannot guarantee reliable state consistency in adversarial IoT environments.

#### 6.8.3. Experimental Results Under Byzantine-Resilient Consensus Mode

The proposed Byzantine-resilient consensus mode introduces adversarial-aware aggregation before blockchain commitment. Sensor observations are first evaluated according to consistency constraints, and potentially corrupted measurements are filtered before contributing to the final state.

**Figure 13:**
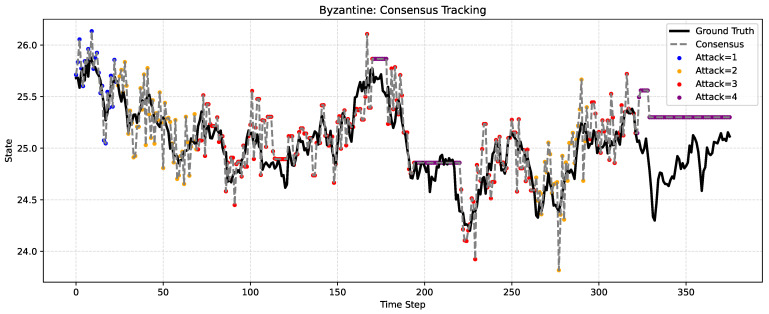
Consensus tracking under Byzantine-resilient aggregation.

**Figure 14:**
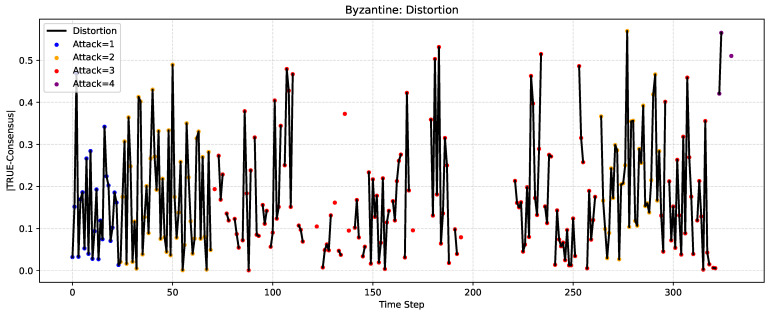
State distortion under Byzantine-resilient aggregation.

Figure 13 and Figure 14 demonstrate that the proposed mechanism maintains stable environmental state estimation under adversarial conditions.

When a small number of nodes are compromised, abnormal observations are effectively isolated before aggregation, preventing malicious measurements from significantly affecting the blockchain-committed state. Consequently, the consensus output remains closely aligned with the ground-truth process.

As the adversarial ratio increases, the system continues to preserve state integrity by prioritizing statistically consistent observations from benign nodes. Unlike Vanilla and Mean aggregation, where corrupted inputs directly distort the consensus output, the Byzantine-resilient mechanism limits adversarial influence through selective acceptance.

Under extremely high attack intensity, the system exhibits a conservative behavior that when insufficient trustworthy observations remain, consensus updates are reduced or temporarily rejected rather than committing potentially corrupted states. This demonstrates an integrity-preserving strategy that favors trustworthy state commitment over continuous availability.

The experimental results confirm that Byzantine-resilient aggregation provides substantially improved robustness against compromised sensing nodes, enabling reliable blockchain-based state coordination in adversarial IoT environments.

### 6.9. Discussion

#### 6.9.1. Quantitative Evaluation

To evaluate the robustness of different aggregation mechanisms under adversarial sensing conditions, we conduct experiments across three representative methods: Vanilla averaging, Mean aggregation, and Byzantine-resilient aggregation. The system is evaluated under increasing attack intensities ranging from 0 to 4, where attack intensity corresponds to the number of compromised sensing nodes among the five non-ground-truth nodes.

Table 3 reports the quantitative results in terms of RMSE, MSE, MAE, bias, standard deviation (STD), and high-order error statistics including median, P95, and maximum error.

The results demonstrate a clear degradation trend across all methods as attack intensity increases. Both Vanilla and Mean aggregation exhibit rapid performance deterioration, with RMSE increasing from approximately 0.18 under no attack to over 4.3 to 4.4 under high-intensity attacks. This indicates strong vulnerability to coordinated adversarial perturbations in simple averaging-based estimators.

In contrast, the Byzantine-resilient aggregation method maintains stable performance under low to moderate attack intensities (1–3), with RMSE remaining within the range of 0.18 to 0.24. This stability is consistent with the theoretical robustness of Byzantine filtering mechanisms, which reduce the influence of compromised nodes through resilient aggregation rules.

However, at attack intensity 4, a sharp performance degradation is observed, where RMSE increases to 2.68. This phenomenon is attributed to the breakdown of the majority assumption, where compromised nodes dominate the sensing population, significantly reducing the effectiveness of robust aggregation.

Beyond average error metrics, higher-order statistics such as P95 and maximum error further highlight the robustness differences among methods. Vanilla and Mean aggregation exhibit significantly larger tail errors under high attack intensity, reaching maximum error values above 8–10. In contrast, Byzantine aggregation maintains relatively bounded error distributions under moderate attacks, indicating improved stability and reduced sensitivity to outliers.

The bias and standard deviation metrics further confirm this trend. Byzantine aggregation consistently achieves lower variance compared to Mean and Vanilla methods under attack intensities 1–3, suggesting improved estimation stability in adversarial environments.

The experimental results validate that robust aggregation mechanisms significantly improve resilience in adversarial sensing environments. While Vanilla and Mean methods degrade monotonically with increasing attack intensity, Byzantine-resilient aggregation exhibits a non-linear degradation pattern, maintaining stability under moderate attacks but failing under extreme corruption scenarios, which behavior aligns with theoretical expectations for majority-based robust estimators in distributed sensing systems.

#### 6.9.2. TEE System Decomposition and Ablation

This subsection provides a detailed clarification of the system decomposition and experimental interpretation in response to concerns regarding (i) the attribution of performance improvements across system components, and (ii) the role and implementation scope of the Trusted Execution Environment (TEE) within the proposed framework. TEE protects execution integrity but does not guarantee sensing correctness.

The proposed framework is designed as a layered architecture consisting of three logically separated components, including (1) a Trusted Execution Environment (TEE) layer, (2) a data integrity layer, and (3) a consensus aggregation layer. In the current deployment, the TEE layer remains fixed across all experiments and therefore is not treated as an independent variable.

In this design, the TEE layer is provided by a third-party TrustZone/OP-TEE-enabled edge infrastructure and remains invariant across all experimental configurations. It serves as a secure execution substrate for consensus-related computations, ensuring that the execution of aggregation logic and blockchain commitment processes is protected against software-level tampering.

Importantly, the TEE layer is not treated as a tunable or configurable variable in this study. Instead, it is considered a fixed infrastructure assumption shared across all experimental modes. Therefore, the observed performance differences across Vanilla, Mean, and Byzantine-resilient configurations are attributed exclusively to variations in the data integrity and consensus aggregation layers. This design choice allows the evaluation to isolate the impact of statistical aggregation and Byzantine-resilient mechanisms under identical trusted execution conditions.

Although explicit factorial ablation across all possible combinations (e.g., filtering-only without TEE, or K-confirmation-only isolation) is not implemented, the experimental design can be interpreted as a controlled comparison over the consensus-layer design space under a fixed trusted execution substrate. Specifically, the evaluated configurations correspond to distinct instantiations of the consensus pipeline, including

1. Vanilla Mode, direct statistical aggregation without adversarial filtering or robustness enhancement;
2. Mean Mode, statistical averaging-based aggregation without outlier suppression;
3. Byzantine-Resilient Mode, robust aggregation with adversarial filtering mechanisms operating within the same trusted execution substrate.

Within this interpretation, the contribution of the proposed system is concentrated in the design of the aggregation and filtering mechanisms, rather than in the trusted execution infrastructure itself. The TEE is therefore treated as an enabling but non-variable component that ensures execution integrity across all experimental settings.

The Trusted Execution Environment used in this work is provided by a third-party edge computing platform supporting TrustZone/OP-TEE functionality. The authors do not implement, modify, or instrument the underlying enclave architecture, nor is access to enclave-level internal execution metrics available in the current deployment environment. Accordingly, the TEE is not evaluated as a standalone experimental factor.

Instead, its role in the proposed system is to ensure the integrity of the execution of aggregation and blockchain commitment logic. However, it is important to emphasize that the TEE does not provide guarantees regarding the correctness or authenticity of sensor-generated data. Compromised or faulty sensing nodes may still inject adversarial or corrupted observations into the system before enclave-level processing. This fundamental limitation motivates the introduction of Byzantine-resilient aggregation and filtering mechanisms at the application layer.

The experimental comparison focuses on the aggregation layer, including Vanilla, Mean, and Byzantine-resilient strategies, in order to isolate their impact on adversarial robustness.

Future work will investigate finer-grained ablation studies by independently evaluating K-threshold confirmation and alternative robust aggregation mechanisms such as median, trimmed mean, and quorum voting.

Based on the above decomposition, the improvements observed in the Byzantine-resilient configuration should be attributed to the joint effect of robust aggregation and adversarial filtering rather than to the presence of the TEE itself. The TEE ensures secure execution of the processing pipeline, while robustness against malicious sensing behavior is achieved at the consensus layer.

Therefore, the experimental comparison should be interpreted as a study of consensus-layer robustness under a fixed trusted execution infrastructure, rather than a comparison of different TEE configurations or implementations.

The proposed evaluation framework isolates the contribution of consensus and aggregation mechanisms under a constant Trusted Execution Environment. This design allows a focused assessment of adversarial robustness in blockchain-enabled IoT systems, while maintaining a consistent and secure execution substrate across all experimental scenarios.

#### 6.9.3. Finality Model and Applicability

To provide intuition regarding confirmation depth and transaction finality, we adopt the classical probabilistic rewriting model

(19) $$P_{\mathrm{rewrite}}\leq {\left(\frac{\alpha }{1-\alpha }\right)}^{k}$$

where α denotes the adversarial fraction and *k* denotes the confirmation depth.

This bound originates from Nakamoto-style blockchain systems and is used solely as an illustrative model for probabilistic finality.

It should not be interpreted as an exact security guarantee for the permissioned agricultural blockchain deployed in this work.

The experimental platform follows a permissioned authority-based architecture whose security primarily depends on validator trust assumptions, quorum formation, and operational governance policies rather than proof-of-work style probabilistic consensus.

Therefore, the rewriting bound is presented only to provide conceptual intuition regarding confirmation depth and should not be viewed as a rigorous characterization of the deployed ledger.

#### 6.9.4. Role and Limitation of Trusted Execution Environments

A common misconception is that a Trusted Execution Environment can directly prevent adversarial sensing attacks. In practice, the TrustZone/OP-TEE infrastructure guarantees execution integrity, memory isolation, and protection of cryptographic assets. However, it does not guarantee that sensor observations entering the trusted execution pipeline are truthful.

Compromised sensing nodes may still generate arbitrary falsified environmental observations before data enters the trusted environment. Consequently, trusted execution alone cannot eliminate sensing-layer adversarial manipulation.

To address this limitation, the proposed framework incorporates Byzantine-resilient aggregation and consensus mechanisms that operate within the trusted execution environment. The TEE ensures that aggregation logic executes faithfully, while adversarial robustness is achieved through robust filtering, statistical consistency analysis, and confirmation policies.

All experimental configurations reported in this study are executed within the same TrustZone/OP-TEE infrastructure. Therefore, the observed performance differences between Vanilla, Mean, and Byzantine modes originate from the aggregation and consensus layer rather than from the trusted execution environment itself. The experimental results indicate that the principal source of adversarial resilience is the Byzantine-resilient aggregation mechanism, whereas the TEE primarily provides trusted execution guarantees for the deployed logic.

## 7. Conclusions and Future Work

### 7.1. Conclusions

This work addresses the problem of trustworthy environmental-state construction in resource-constrained smart agriculture IoT systems, where sensor observations are inherently noisy and potentially adversarial. Instead of treating trust as an abstract assumption, we design and implement a complete system that integrates Trusted Execution Environments (TEEs), Byzantine-robust aggregation, and a lightweight permissioned blockchain to support verifiable environmental-state consensus.

The key design principle of the proposed framework is to decouple and explicitly coordinate three trust layers: execution integrity at the edge, data reliability through robust aggregation, and state-level consistency via blockchain commitment. In this architecture, TEEs provide hardware-assisted protection for preprocessing and attestation of sensor data, ensuring that data entering the system originates from verified execution contexts. On top of this, a Byzantine-resilient aggregation mechanism filters corrupted or manipulated sensor readings and constructs a consistent environmental state from distributed observations. Finally, a permissioned blockchain layer records only validated state commitments, providing tamper-evident and traceable system-level state evolution.

Unlike traditional blockchain–IoT systems that directly record raw sensor data or rely solely on cryptographic integrity, the proposed framework focuses on ensuring consistency of the derived environmental state under adversarial sensing conditions. Blockchain is therefore used as a coordination and finality layer rather than a transactional ledger, enabling lightweight consensus suitable for IoT-scale deployments.

Experimental results obtained from a real greenhouse IoT prototype demonstrate that the proposed architecture significantly improves robustness under sensor corruption and adversarial perturbation. In particular, compared to a baseline without Byzantine aggregation, the system maintains stable state estimation and consistent blockchain commitments even under increased levels of sensing interference. The results further show that TEE-assisted preprocessing improves resistance to gateway-level compromise, while the combination with robust aggregation prevents corrupted observations from propagating to the consensus layer.

An important observation from the experiments is that system reliability is maintained through adaptive commitment behavior. When the level of sensing uncertainty exceeds the aggregation tolerance, the system naturally reduces or suspends state commitment, preventing corrupted environmental states from being recorded on-chain. This highlights the role of consensus as a safety-preserving mechanism rather than a purely continuous data logging process.

### 7.2. Limitations and Future Work

The current prototype is evaluated in a controlled greenhouse environment, which may not fully reflect the scale and heterogeneity of large industrial agricultural deployments. Future work will focus on scaling the system to large-scale sensor networks with dynamic topology and heterogeneous device capabilities.

The adversarial model considered in this study primarily focuses on data-level manipulation and partial node compromise. More advanced threats, such as coordinated multi-node collusion, adaptive adversarial behavior, and cross-layer attack strategies, remain to be investigated.

In addition, the current consensus layer adopts a lightweight permissioned design suitable for edge environments. Future research may explore hierarchical consensus architectures that combine edge-level state agreement with higher-level regional coordination.

Finally, this work opens a direction for integrating trustworthy sensing, distributed estimation, and blockchain-based coordination into a unified framework for cyber–physical systems beyond agriculture, including environmental monitoring and industrial IoT applications.

## Figures and Tables

**Table 1:** Notation Summary.

Symbol	Definition	Meaning
$B=(B_{1},\ldots ,B_{k})$	Blockchain sequence	Ordered sequence of blocks
$B_{i}=\left(D_{i},h_{i-1},\sigma_{i}\right)$	Block *i*	Contains data $D_{i}$, previous hash $h_{i-1}$, and signature $\sigma_{i}$
$B_{i}^{\prime },B^{\prime }$	Modified block/blockchain	Altered versions used in immutability analysis
h(·)	Cryptographic hash function	Collision-resistant hash function
$h(B_{i})$	Hash of block $B_{i}$	Cryptographic digest of block content
$h_{i-1}$	Previous block hash	Links current block to predecessor
$D_{i}$	On-chain data	Data contained in block $B_{i}$
$D_{i}^{\mathrm{off}}$	Off-chain data object	External sensor, edge, or monitoring data
$D_{i}^{\mathrm{off}*}$	Modified off-chain data	Tampered version of $D_{i}^{\mathrm{off}}$
$h_{\mathrm{on}\text{-}\mathrm{chain}}$	On-chain hash commitment	Hash recorded on-chain for verification
Detect(·)	Tampering detection function	Verifies consistency between off-chain data and on-chain commitments
1,0	Detection output	1: integrity verified; 0: tampering detected
M	Turing machine	Abstract computational model
*f*	Computable function	Function computable by Turing machine
$M_{f}$	Turing machine for *f*	Turing machine computing function *f*
$C_{M}$	BVM contract	Smart Contract as Turing machine
$\sigma_{i}$	Digital signature	Cryptographic authentication signature
A	PPT adversary	Probabilistic polynomial-time attacker
λ	Security parameter	computings cryptographic hardness
negl(λ)	Negligible function	Function vanishing faster than inverse polynomials
α	Adversarial fraction	Fraction of mining or staking power computing by adversary
$P_{\mathrm{rewrite}}$	Rewriting probability	Probability adversary rewrites blockchain history
*N*	Number of nodes	Total participating blockchain nodes
*L*	Blockchain length	Total number of blocks in the chain

**Table 2:** Comparison with Existing Trust Architectures in IoT and Blockchain Systems.

Work	TEE	Byzantine Aggregation	Blockchain	State Consensus
TEE-Accelerated BFT Systems [20,23,24]	✓	✓	✓	×
TEE-Blockchain Systems [22,29,48]	✓	×	✓	×
Byzantine Consensus Systems [26,27,28]	×	✓	✓	×
End-to-End TEE Systems [29,31,32]	✓	×	×	×
**Proposed System**	✓	✓	✓	✓

**Note:** ‘✓’ denotes the presence of the feature, while ‘×’ indicates its absence.

**Table 3:** Quantitative Performance Comparison under Different Attack Intensities.

Mode	Intensity	RMSE	MSE	MAE	Bias	STD	P95	MaxError	CI95
Byzantine	1	0.184	0.034	0.147	0.043	0.179	0.333	0.468	0.046
Byzantine	2	0.242	0.058	0.200	0.049	0.237	0.422	0.570	0.031
Byzantine	3	0.216	0.047	0.168	0.043	0.211	0.459	0.674	0.019
Byzantine	4	2.680	7.182	0.876	0.870	2.535	0.986	12.532	0.529
Mean	0	0.186	0.035	0.149	0.048	0.180	0.402	0.466	0.029
Mean	1	1.592	2.535	1.201	0.276	1.568	3.280	3.686	0.302
Mean	2	2.381	5.670	1.962	1.223	2.043	4.606	5.604	0.399
Mean	3	3.507	12.298	2.896	2.117	2.795	6.344	8.274	0.428
Mean	4	4.436	19.678	3.774	3.071	3.201	7.785	10.509	0.535
Vanilla	0	0.175	0.031	0.146	0.079	0.157	0.314	0.331	0.039
Vanilla	1	1.761	3.101	1.427	0.873	1.530	3.304	3.381	0.295
Vanilla	2	2.258	5.097	1.833	1.179	1.925	4.571	5.563	0.323
Vanilla	3	3.425	11.730	2.738	2.026	2.761	6.342	8.051	0.443
Vanilla	4	4.309	18.567	3.554	3.210	2.874	8.332	9.323	0.737

## Data Availability

All content presented in this manuscript is free of intellectual property restrictions and does not conflict with any current or pending commercial interests. The dataset and source code used in this study are not publicly available due to institutional policies and the ongoing commercialization of the system. However, access to the dataset and relevant portions of the codebase can be granted upon reasonable request for academic and non-commercial research purposes. Requests for access may be directed to the technical contact at lilanlan@chzc.edu.cn.
